# Orthodontic Wire Ingestion during Treatment: Reporting a Case and Review the Management of Foreign Body Ingestion or Aspiration (Emergencies)

**DOI:** 10.1155/2013/426591

**Published:** 2013-06-18

**Authors:** Mohammad Hoseini, Seyed Morteza Saadat Mostafavi, Navid Rezaei, Ehsan Javadzadeh Boluri

**Affiliations:** ^1^Department of Orthodontics and Dentofacial Orthopedics, School of Dentistry, Shahid Sadoughi University of Medical Sciences, Daheye Fajr BLV, Imam Avenue, Yazd 89195/165, Iran; ^2^School of Dentistry, Shahid Sadoughi University of Medical Sciences, Daheye Fajr BLV, Imam Avenue, Yazd 89195/165, Iran

## Abstract

Today orthodontic treatment is in growing demand and is not limited to a specific age or social group. The nature of orthodontic treatment is such that the orthodontic wires and appliances, which are used to apply force and move the teeth, are exposed to the oral cavity. Shaping and replacing these wires in oral cavity are the major assignments of orthodontist on appointments. Therefore, we can say that orthodontic treatment requires working with dangerous tools in a sensitive place like oral cavity which is the entrance of respiratory and digestive systems. In this paper, a case of ingesting a broken orthodontic wire during eating is reported, and also necessary remedial measures at the time of encountering foreign body ingestion or aspiration are provided.

## 1. Introduction

Optional or accidental foreign body ingestion is common. Although in most cases these objects are excreted, in 1% of the cases, complex problems such as gastrointestinal perforation are seen [1, 2] which can sometimes lead to serious risks, including death. In the United States, 1500 people die each year due to foreign body ingestion [3, 4]. Incidence of ingesting dental materials and appliances varies in different studies. It was 3.6% to 27.7% in Tamura's review paper, and the majority belonged to adults. Aspiration or ingestion of orthodontic appliances is less common and depends on the kind of appliance [5]. Orthodontic appliances are usually small and are difficult to use especially when covered with saliva. The risk of objects to fall back into oropharynx and ingested or aspirated is more when the patient is in supine position, and it gets worse if you break the appliance. Depending on the shape, size, and flexibility of the object, some events may have minimal risk, while some may even be fatal. Prevention is the best method, but when happened, an efficient management of the event would be critical to save the patient's life [6]. The aim of this paper is to present a case of orthodontic wire ingestion and consequences and suggests approaches in the face of these events.

## 2. Case Report

**Figure 1:**
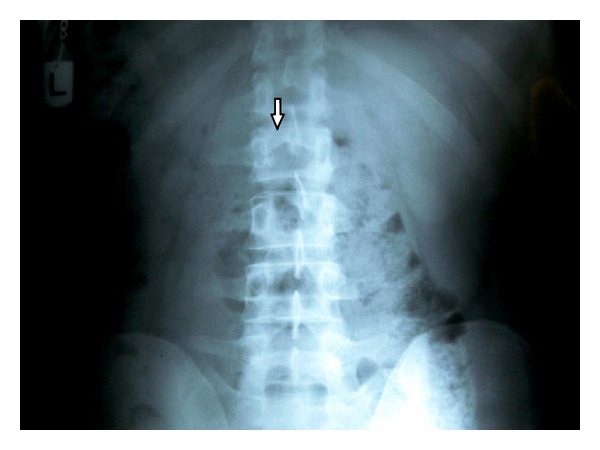
Abdominal radiographic image: presence of orthodontic wire in stomach location.

The patient is a 29-year-old man who had been treated at a private clinic in the city (Yazd, Iran). Nonextraction treatment plan with 0.18 Roth system (Dentaurum Germany) was accomplished for the patient, and 0.16 Ni Ti arch wire (3M Unitek) was placed, at the finishing phase, on the maxillary teeth. At this time, due to lack of patient compliance, the brackets on left first and second premolars on maxillary arch were debonded. When eating, patient noticed that a piece of wire was ingested and at the same time a gastric irritation was felt. This filling was repeated the following day. The patient referred to a GI specialist. Radiographic images were taken to track and locate the ingested wire (Figure 1). The wire was found at the lower stomach.

**Figure 2:**
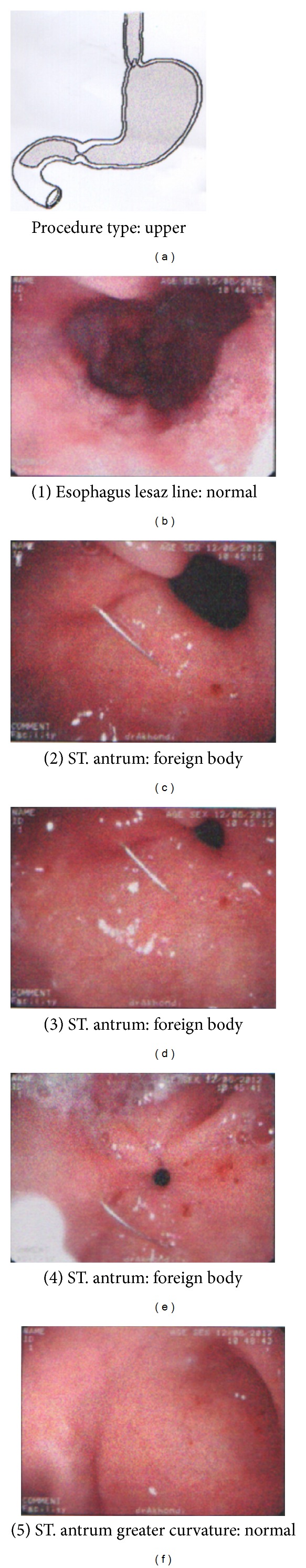
Endoscopic images of orthodontic wire in antrum of stomach before and after endoscopy.

The wire was removed from the stomach under sedation with Midazolam in Shahid Sadooghi Hospital, Yazd, Iran (Figure 2).

Treatment was performed by endoscope including an overtube to prevent esophagus being ruptured by the wire. However, due to relatively large size of the wire, there was some degree of esophageal scratches that required IV chemotherapy (Metronidazole, ceftriaxone, Pantoprazol) with a 24 hr NPO. Burning sensation continued for a while. Treatment was terminated in 3 months.

## 3. Discussion

Foreign body ingestion and aspiration are potentially life threatening emergencies that may happen in any field of dentistry [1, 2]. Preventing from ingestion and aspiration is the best method and is achievable through following the principals of prevention [3].

Orthodontic wires may break in two situations. First, when the patient is sitting on the dental chair and orthodontist is cutting the end of a newly placed arch wire. Although there are customized end cutting players that hold the cut end, but it may happen for any reason such as using inefficient or inappropriate instrument. Second, as in this case, the patient is discharged, and in everyday life and activities the wire breaks. This may happen where a part of arch wire, due to treatment plan, is unsupported and is exposed to masticatory forces, or negligence in mastication and applying too much force lead to debonding some of brackets.

If a foreign body remains in oropharynx, the patient should be placed in reclined position and encouraged to cough deeply. Immediate priority is to ensure that the airways are clear, if they are not, immediate recognizable signs will appear soon. Orthodontists and general dentists should be able to recognize the signs and symptoms of airway obstruction including choking, inspiratory stridor, and using accessory respiratory muscles to breath. According to Mayo clinic website, the common sign is gripping throat with hands. If the patient did not show this sign, it would be necessary to look for inability to speak; difficulty in breathing; respiratory stridor; inability to cough; blue discoloration on skin, lips, and nails; and loss of consciousness [7]. If deep cough had no benefit, Heimlich maneuver should be performed to open the airways [8]. It is necessary to be successful very quickly. Otherwise, you must notify and summon urgent medical care team. Meanwhile, life support measures such as cricothyroidotomy, if indicated, should be taken to open the airway [9]. We can also make use of bronchoscopy to retrieve foreign bodies within the trachea and bronchial tree.

If there was no issue with the airways, the oral cavity should be inspected to find the foreign body. If you could not find the object, the patient would be informed in relation to the problem. You refer the patient to hospital for taking X-ray and clinical exam [10]. In case of foreign body ingestion, the patient may experience some problems in digestive tract including obstruction consequent perforation, and abscess formation; bleeding and fistulation; and also the object may remain motionless [11]. Obstruction is more likely in the upper esophagus [12]. This may lead to esophageal perforation with secondary mediastinitis. Esophageal obstruction may be in association with aspiration. So, the foreign body must be removed quickly using a fibro-optic endoscope.

If the foreign body reaches the stomach, it will pass through the digestive tract, with a probability of greater than 90%. It may take 10 days [13].

The risk of perforation or obstruction depends on the size and shape of the object. It is higher for a sharp object. In addition, an object longer than 5 cm is unlikely to pass through the duodenum [14, 15]. When an object leaves the stomach into small intestine, it is likely to pass it. The most common location for perforation or obstruction is ileocaecal valve [11, 16]. The risk of obstruction increases with anatomical anomalies such as constrictions and roughness. Removing the object by means of colonoscopy is possible.

Managing a patient who has ingested an object is accomplished with serial radiographic assessment. Patient must be examined and assessed regarding the clinical symptoms of perforation and obstruction such as pain and nausea and also the signs such as tenderness and guarding. There is no evidence that laxatives are useful and may even increase the risk of perforation [12].

Radiographs are useful to confirm the presence of foreign body, assess the size and shape, determine the location, and also to define the moving in or exiting out of the digestive system. If the patient showed the signs of perforation or the object remained for 2 weeks or more, surgical intervention is required.

Orthodontists and general dentists must be able to recognize the signs of airway obstruction due to remaining foreign body in esophagus.

## 4. Conclusion

In addition to this fact that general dentists and orthodontists must provide precautionary measures to prevent from foreign body aspiration and ingestion, they may be informed how to manage these cases.

One of the necessary measures is preparing chair side emergency equipments. If the airway is open, the patient should be taken to hospital, clinical and radiographic examinations should be performed by qualified persons, and then appropriate treatment should be performed according to diagnosis and location of the foreign body.
